# Triple-Negative Breast Cancer: A Review of Current Curative Intent Therapies

**DOI:** 10.3390/curroncol29070378

**Published:** 2022-07-07

**Authors:** Isaiah MacDonald, Nancy A. Nixon, Omar F. Khan

**Affiliations:** 1 Faculty of Medicine and Dentistry, University of Alberta, Edmonton, AB T6G 2R7, Canada; isaiah.macdonald@albertahealthservices.ca; 2 Department of Oncology, University of Calgary, Calgary, AB T2N 4N2, Canada; nancy.a.nixon@ahs.ca

**Keywords:** breast cancer, triple negative, adjuvant, neoadjuvant, curative intent, chemotherapy, immunotherapy, PARPi

## Abstract

Breast cancer is the most commonly diagnosed malignancy in women, with triple-negative breast cancer (TNBC) accounting for 10–20% of cases. Historically, fewer treatment options have existed for this subtype of breast cancer, with cytotoxic chemotherapy playing a predominant role. This article aims to review the current treatment paradigm for curative-intent TNBC, while also reviewing potential future developments in this landscape. In addition to chemotherapy, recent advances in the understanding of the molecular biology of TNBC have led to promising new studies of targeted and immune checkpoint inhibitor therapies in the curative-intent setting. The appropriate selection of TNBC patient subgroups with a higher likelihood of benefit from treatment is critical to identify the best treatment approach.

## 1. Introduction

In Canada, 28 900 new cases of breast cancer are projected in 2022, representing over 25% of all new cancer diagnoses in women [1]. Triple-negative breast cancer (TNBC) accounts for approximately 10–20% of all cases [2]. TNBC is associated with aggressive tumor phenotypes and a higher risk of recurrence (particularly within the first three to five years after diagnosis) despite higher initial response rates to curative-intent treatment [3]. In the metastatic setting, survival outcomes are much worse than hormone-receptor and/or HER-2-positive breast cancers [2]. Historically, treatment options for TNBC were limited to cytotoxic chemotherapy. However, TNBC represents a highly heterogenous group of tumors, and understanding the molecular biology of a specific tumor allows for personalized treatment (in turn, minimizing unnecessary treatment toxicity while improving cancer outcomes) [4]. This article reviews the current landscape of curative-intent TNBC treatment strategies, in addition to looking ahead toward future directions and possible therapeutic approaches. Treatment options are summarized for adjuvant (Table 1) and neoadjuvant (Table 2) TNBC, and drug classes and mechanisms of action are detailed in Table 3. 

## 2. Cytotoxic Chemotherapy

TNBC shows initial sensitivity to cytotoxic chemotherapy, with higher response rates observed compared to other breast cancer subtypes [3]. Approximately 30–40% of TNBC cases will achieve a pathologic complete response (pCR) after treatment with a third-generation sequential anthracycline and taxane-based chemotherapy regimen [27], with pCR appearing to provide a valid surrogacy for recurrence-free and overall survival after treatment with chemotherapy in the TNBC subtype [28,29]. Superiority of the treatment with an anthracycline and taxane (over an anthracycline-sparing regimen) was established in the ABC group of trials (combining the USOR 06-090, NSABP B-46/USOR 07,132, and NSABP-B49 studies), which failed to demonstrate noninferiority of the anthracycline-sparing regimen [5]. Subgroup analysis of this study showed absolute four-year invasive disease-free survival benefits of 2.5% in node-negative TNBC patients, 10.9% in TNBC patients with N1 disease (1–3 lymph nodes involved), and 11.0% in TNBC patients with four or more lymph nodes involved. However, wide confidence intervals were noted in each of these subgroups, with the hazard ratio crossing 1.0. 

### 2.1. Capecitabine

The value of pCR as a surrogate endpoint for survival in TNBC patients treated with chemotherapy led to studies assessing the impact of treatment escalation in patients where a pCR was not achieved. In the CREATE-X clinical trial, 910 patients with HER-2-negative breast cancer and without a pCR after neoadjuvant chemotherapy were randomized to standard care with or without capecitabine for between six and eight cycles of treatment [6]. While the overall study population benefited from capecitabine, the improvement in outcomes was exclusively driven by the TNBC subgroup, for both disease-free survival (69.8% versus 56.1%, HR 0.58, 95% CI 0.39–0.87) and overall survival (78.8% versus 70.3%, HR 0.52, 95% CI 0.30–0.90). Conversely, the GEICAM/2003-11_CIBOMA/2004-01 trial (which also compared capecitabine in the adjuvant setting to observation) failed to show an improvement in disease-free survival (79.6% versus 76.8%, HR 0.82, 95% CI 0.63–1.06) or overall survival (86.2% versus 85.9%, HR 0.92, 95% CI 0.66–1.28) [7]. However, this study also included patients with a pCR after neoadjuvant therapy (a lower-risk group), potentially accounting for the difference in efficacy. A systematic review and meta-analysis of capecitabine usage in the neoadjuvant and adjuvant settings showed significant improvements in disease-free survival (HR 0.75, 95% CI 0.65–0.86, *p* < 0.001) and overall survival (HR 0.63, 95% CI 0.53–0.77, *p* < 0.001) [30]. 

The SYSUCC-001 clinical trial also assessed the role of adjuvant capecitabine therapy, but at a lower dose (650 mg/m^2^ twice daily) given continuously for one year as maintenance therapy [8]. This trial randomized 424 patients with TNBC to capecitabine versus observation, showing an improvement in five-year disease-free survival (82.8% versus 73.0%, HR 0.64, 95% CI 0.42–0.95, *p* = 0.03), but without a statistically significant difference in five-year overall survival (85.5% versus 81.3%, HR 0.75, 95% CI 0.47–1.19, *p* = 0.22).

### 2.2. Platinum Agents

Platinum cytotoxic agents (such as carboplatin or cisplatin) cause DNA strand breaks via cross-linkage of DNA strands [31], increasing their effectiveness in tumors with impaired DNA repair pathways (a finding commonly seen in *basal-like-1* TNBC [32,33], as well as in patients with hereditary mutations such as *BRCA* or other homologous recombination pathway genes [34]). Therefore, multiple studies have focused on the role of platinum-based chemotherapy for TNBC, with mixed results. In the GeparSixto clinical trial, the addition of carboplatin to neoadjuvant therapy resulted in a pCR in 53.2% of TNBC patients (84/158), compared to 36.9% (58/157) without carboplatin (*p* = 0.005) [13]. Similarly, in the CALGB 40,603 and BrighTNess studies, early-stage TNBC patients had higher pCR rates with the addition of carboplatin [14,15]. A post hoc analysis of BrighTNess also showed improvements in event-free survival rates with the addition of carboplatin to neoadjuvant chemotherapy (HR 0.57, *p* = 0.02), although no difference in overall survival outcomes was seen [16]. One meta-analysis of nine randomized controlled trials confirmed a significant increase in pCR rate with platinum-based neoadjuvant therapy (52.1% versus 37.0%, *p* < 0.001) [35]. 

In the adjuvant setting (particularly after neoadjuvant treatment with an anthracycline and taxane), the role of platinum agents is less clear. The EA1131 study [9] randomized patients with residual disease after neoadjuvant therapy to receive either capecitabine or platinum-based chemotherapy, and failed to demonstrate noninferiority of the platinum agent. The three-year event-free survival in both arms of this study was much worse than anticipated (42% in the platinum chemotherapy arm, versus 49% in the capecitabine arm). This is considered to relate to a higher proportion of basal-like tumors (78%) in the EA1131 study, or a higher burden of residual disease compared to the CREATE-X trial. The ongoing NRG BR-003 clinical trial (NCT02488967) [36] is randomizing higher-risk patients (with either node-positive disease or a tumor size of at least 3.0 cm) not treated in the neoadjuvant setting to receive standard adjuvant anthracycline/taxane combination chemotherapy with or without carboplatin. This study is estimated to have a primary completion date in November 2023 and will provide important insight into the role of platinum therapy in the adjuvant setting. A recent meta-analysis (looking at 2425 patients across eight clinical trials in the neoadjuvant and adjuvant settings) showed improvements in disease-free survival and overall survival with the addition of carboplatin to an anthracycline/taxane backbone, both with trial-level and individual patient-level analyses [37]. Overall, at this time, outside of clinical trials, the role of platinum chemotherapy in TNBC is primarily restricted to the neoadjuvant setting. 

## 3. Targeted Therapies

### PARP Inhibitors

Poly-ADP-ribose polymerases (PARP) are a group of diverse enzymes involved in DNA damage repair of single-stranded breaks, amongst other cellular functions [38]. Double-stranded DNA breaks are repaired through multiple mechanisms, including homologous recombination, but in the setting of an impairment in these pathways (such as that seen with mutations in the *BRCA1* or *BRCA2* genes, for example), PARP plays a more critical role. In patients with mutations in *BRCA1/2* (and, to a lesser extent, other homologous recombination pathway genes as well), the inhibition of PARP leads to the inhibition of an effective DNA damage response, and subsequently cell death through a process known as synthetic lethality [39].

The role of PARP inhibitors in the neoadjuvant setting currently remains limited. The I-SPY-2 adaptive randomized phase II trial estimated an 88% predicted probability of success for the addition of veliparib plus carboplatin for neoadjuvant therapy in TNBC, with a pCR rate of 51% versus 26% in the control group (anthracycline/taxane chemotherapy) [17,18]. This led to the development of the phase III BrighTNess trial, which randomized patients to three arms—standard treatment (paclitaxel followed by doxorubicin/cyclophosphamide), standard treatment plus carboplatin, or standard treatment plus carboplatin and veliparib. As noted above, while the addition of carboplatin improved pCR [15] and event-free survival [16] rates compared to standard therapy, the veliparib arm did not provide any additional benefit, and increased toxicity. However, this study was not restricted to patients with *BRCA* mutations, potentially accounting for the negative result. A single-arm, nonrandomized phase II study of talazoparib (NEOTALA) assessed patients with germline *BRCA1/2* mutations and locally advanced HER-2-negative breast cancer (tumor size at least 1.5 cm) [19]. Patients were treated with talazoparib 1 mg daily for 24 weeks prior to surgery, followed by adjuvant therapy based on investigator choice. The results of this study were presented at the 2021 American Society of Clinical Oncology Annual Meeting, with a reported pCR rate of 49.2% in the intention-to-treat population based on independent central review (a pCR rate comparable to those expected from anthracycline/taxane chemotherapy combinations, despite these patients not having received neoadjuvant cytotoxic therapy). Larger studies in the *BRCA1/2*-mutated population are needed to clarify the role of PARP inhibition and cytotoxic chemotherapy in neoadjuvant TNBC. 

Conversely, in the adjuvant setting, PARP inhibition plays a critical role. The international phase III, double-blinded, multi-center OlympiA clinical trial [10] assessed the role of adjuvant olaparib therapy in patients with germline *BRCA1/2* mutations and high-risk HER-2-negative breast cancer (defined as having lymph node involvement or tumor size greater than 2.0 cm if not treated with neoadjuvant therapy, or the presence of residual disease if treated with neoadjuvant therapy). Patients were assigned to one year of olaparib (300 mg twice daily) or placebo therapy, with treatment started within 12 weeks of completion of locoregional therapy. Although hormone-receptor-positive tumors were also allowed on this study, over 80% of the patients included had TNBC. The results of the OlympiA study demonstrated a significant improvement in invasive disease-free survival at three years with olaparib compared to placebo (85.9% versus 77.1%, HR 0.58, 99.5% CI 0.41–0.82, *p* < 0.001), as well as distant disease-free survival (87.5% versus 80.4%, HR 0.57, 99.5% CI 0.39–0.83, *p* < 0.001). While fewer deaths were reported in the olaparib group compared to placebo, the between-group difference did not cross the boundary for significance at the first interim analysis. However, an update presented at the European Society of Medical Oncology Virtual Plenary in March 2022 confirmed an improvement in overall survival, with a 3-year survival of 92.0% with olaparib compared to 89.1% with placebo (HR 0.68, 98.5% CI 0.47–0.97, *p* = 0.009) [11]. Of note, the OlympiA study did not allow TNBC patients with residual disease after neoadjuvant therapy to receive adjuvant capecitabine therapy. As a result, there is a lack of clarity as to the utility of capecitabine in these patients, and further studies are needed to address this. 

## 4. Immunotherapy

The role of immunotherapy in treating curative-intent TNBC is rapidly evolving. Given the potential toxicities associated with checkpoint inhibitor therapy (some of which can be persistent, debilitating, or even fatal), risks and benefits must be carefully considered, particularly in the adjuvant setting [40].

### 4.1. Neoadjuvant Therapy

The only current US Federal Drug Administration (FDA) approval for immunotherapy in early-stage TNBC is for pembrolizumab [41], given in the neoadjuvant and adjuvant setting, based on the results of the KEYNOTE-522 clinical trial. In this trial, patients with stage II/III TNBC were randomized to receive either pembrolizumab or placebo for eight cycles in the neoadjuvant setting (given concurrently with paclitaxel and carboplatin, followed by anthracycline and cyclophosphamide chemotherapy) [20]. After surgery, pembrolizumab or placebo was continued every three weeks for up to nine additional cycles. This trial had two primary endpoints, including pathologic complete response rate (pCR) and event-free survival. At the first interim analysis (consisting of the first 602 patients randomized), an absolute improvement of 13.6% (*p* < 0.001) was seen in pCR rate with pembrolizumab-chemotherapy (64.8%) versus placebo-chemotherapy (51.2%). More recent data from 1174 randomized patients showed an absolute improvement in event-free survival (disease progression precluding surgery, local/distant recurrence, second primary cancer, or death from any cause) of 7.7% (HR 0.63, *p* < 0.001) with pembrolizumab-chemotherapy (84.5%) vs. placebo-chemotherapy (76.8%) [42].

Atezolizumab was studied in the neoadjuvant setting for patients with stage II/III TNBC in the Impassion031 study [21]. In this study, patients received nab-paclitaxel followed by dose-dense doxorubicin and cyclophosphamide, either with atezolizumab or placebo. This was followed by adjuvant atezolizumab to complete 12 months of treatment. The pCR rate with atezolizumab was 58%, compared to 41% in the placebo arm (in patients with PD-L1-positive tumors, the pCR rate was 69% for atezolizumab, compared to 49% for placebo).

In the phase II GeparNUEVO study [43], patients with TNBC with a tumor size of 2 cm or greater were randomized to receive durvalumab versus placebo as a single dose two weeks prior starting neoadjuvant chemotherapy (window-of-opportunity phase). This was followed by durvalumab versus placebo in combination with neoadjuvant chemotherapy (nab-paclitaxel for 12 weeks, followed by dose-dense epirubicin and cyclophosphamide for four cycles). In total, 174 patients were randomized, of which 36.4% had stage I tumors. However, after 117 patients were recruited onto the study, the Independent Data Monitoring Committee recommended eliminating the window-of-opportunity portion of the study, due to delays in receiving systemic chemotherapy. In the overall population, the pCR rate with durvalumab-chemotherapy was 53.4%, compared to 44.2% with placebo-chemotherapy (OR 1.45, *p* = 0.224). However, in patients treated in the window-of-opportunity phase, the pCR rate was 61.0% with durvalumab, compared to 41.4% with placebo. Additional data from GeparNUEVO presented at ASCO 2021 showed significant improvements in the 3-year invasive disease-free survival (84.9% vs. 76.9%, HR 0.54, *p* = 0.0559) and 3-year overall survival with durvalumab versus placebo (95.1% vs. 83.1%, HR 0.26, *p* = 0.076) [22]. The findings of this study raise several important questions regarding the role of immunotherapy in curative-intent TNBC, including regarding the necessity of adjuvant immunotherapy, as well as the utility of pCR as a surrogate endpoint for checkpoint inhibitors (given a modest improvement in pCR but a substantial improvement in overall survival). More information regarding the appropriateness of pCR as a surrogate endpoint in neoadjuvant immunotherapy TNBC trials may come from the NeoTRIP Michelangelo study [44]. This study is assessing carboplatin and nab-paclitaxel chemotherapy with or without atezolizumab in the neoadjuvant setting (all patients received anthracycline chemotherapy in the adjuvant setting). Preliminary analysis of this study showed no improvement in pCR rates with the addition of atezolizumab, but the primary outcome for this trial is event-free survival, and these results are still pending. Additionally, the sequencing of immunotherapy and chemotherapy requires further research, as the benefit of durvalumab in achieving pCR was seen primarily in patients treated in the window-of-opportunity phase.

### 4.2. Adjuvant Therapy

Data supporting the use of immune checkpoint inhibitors in the adjuvant setting are sparse, but multiple trials are currently underway. The SWOG S1418/BR-006 study is comparing one year of pembrolizumab vs. observation after completion of all other standard adjuvant therapies for TNBC with residual malignancy (either tumor size greater than 1 cm or positive lymph nodes) after neoadjuvant chemotherapy [12]. The Impassion-030 (ALEXANDRA) study is comparing adjuvant chemotherapy with or without atezolizumab in patients with stage I-II TNBC [45]. Overall, the use of adjuvant immunotherapy for TNBC should be restricted to the clinical trial setting at this time.

## 5. Future Directions

### 5.1. Circulating Tumor DNA (ctDNA)

Circulating tumor DNA is released following cancer cell death and presents an enticing target to assess disease response and activity [46]. Recent meta-analysis data showed that ctDNA detection after treatment for early breast cancer is associated with a significant reduction in disease-free survival (HR 8.32, 95% CI 3.01–22.99, *p* < 0.01) [47]. Additionally, another study in TNBC assessed 33 patients with residual disease after neoadjuvant therapy [48]. ctDNA was detected in four of these patients, all of whom recurred within nine months of surgery. However, an additional 10 patients ultimately had disease recurrence without detectable ctDNA, leading to a calculated specificity of 100% but a sensitivity of only 33% in this small population. A large phase III randomized trial (ZEST, NCT04915755) [49] is currently recruiting and will provide critical insights into the role of ctDNA. This trial is enrolling patients with TNBC or *BRCA1/2* mutations after completion of standard neoadjuvant and/or adjuvant therapies. Patients are screened using a personalized ctDNA assay, and patients with detectable ctDNA are randomized to receive either niraparib (a PARP inhibitor) versus placebo. If positive, this trial would confirm not only the prognostic value of ctDNA assays, but also their predictive value in the personalization of treatment escalation—a truly practice-changing finding. 

### 5.2. Other Targeted Pathways

Studies are ongoing to assess the role of other targeted treatments in TNBC, including in the curative-intent setting. The phase II randomized FAIRLANE study assessed neoadjuvant ipatasertib versus placebo in combination with paclitaxel chemotherapy for 12 weeks in patients with TNBC with a tumor size of at least 1.5 cm. In the overall population, there was no improvement in pCR rate with the addition of ipatasertib [23]. However, in the subgroup of tumors with mutations in the PIK3CA/AKT1/PTEN pathway, a pCR rate of 39% with ipatasertib was seen, compared to just 9% in the placebo arm. Another potential treatment strategy is the androgen receptor (AR), which is expressed in many TNBC patients, although the data supporting the use of treatments such as enzalutamide or abiraterone are extremely limited and primarily only in the metastatic setting [24]. Therefore, these treatments should not be used in the curative-intent setting outside of a clinical trial at this time. 

### 5.3. Antibody–Drug Conjugates (ADCs)

While there are no currently approved antibody–drug conjugates in the curative-intent setting for TNBC, several molecules have demonstrated activity in the metastatic setting. These include sacituzumab govitecan (an ADC consisting of an antibody targeting the human trophoblast cell-surface antigen 2 (Trop-2) linked to SN-38, a potent topoisomerase inhibitor) [25], as well as trastuzumab deruxtecan (an ADC consisting of an antibody targeting the HER-2 receptor linked to another topoisomerase inhibitor, deruxtecan). Recent data showed an impressive efficacy of trastuzumab deruxtecan in previously treated metastatic TNBC with a low expression of HER-2 (defined as 1+ on immunohistochemistry (IHC) or 2+ on IHC with negative in situ hybridization testing) [26]. A phase III clinical trial of sacituzumab govitecan in patients with high relapse risk after standard neoadjuvant therapy (NCT04595565) is currently recruiting. At this time, ADCs should not be used for curative-intent TNBC treatment outside of a clinical trial.

## 6. Conclusions

In summary, patients with TNBC have more curative-intent treatment options than ever before. While cytotoxic chemotherapy continues to play a critical role in TNBC management, new targeted and immunotherapy options are creating seismic shifts in the approach to managing patients. Additional research is needed to better understand appropriate sequencing, and escalation and de-escalation of therapies.

## Figures and Tables

**Table 1:** Adjuvant Triple Negative Breast Cancer (TNBC) curative-intent treatment.

	Trial (NCT Number)	Phase	Stage	Treatment	Patients in Analysis	Outcomes	Refs
Chemotherapy	ABC Trials (NCT00493870, NCT00887536, NCT01547741)	III	Early II– III	Docetaxel and cyclophosphamide versus doxorubicin and cyclophosphamide	1288	DFS: HR 1.42, 95% CI 1.04–1.94 OS: not significant (favoring AC)	[5]
	CREATE-X (NCT00130533)	III	Early I–IIIb	Patients without pCR, standard of care with or without capecitabine	286	DFS: HR 0.58, 95% CI 0.39–0.87 OS: HR 0.52, 95% CI 0.30–0.90	[6]
	GEICAM/2003-11_CIBOMA/2004-01 (NCT00130533)	III	Early I–III	Capecitabine versus observation	869	DFS: HR 0.82, 95% CI 0.63–1.0 OS: HR 0.92, 95% CI 0.66–1.28	[7]
	SYSUCC-001 (NCT01112826)	III	Early I–III	Low-dose capecitabine vs. observation	424	DFS: HR 0.64, 95% CI 0.42–0.95 OS: HR 0.75, 95% CI 0.47–1.19	[8]
	EA1131 (NCT02445391)	III	Early II–III	Neoadjuvant chemotherapy followed by adjuvant capecitabine versus platinum	308	DFS: 49.4%, 95% CI 39.0 to 59.0 versus 42.0%, 95% CI 30.5–53.1 DFS: HR 1 (Ref) versus 1.06, 95% CI 0.62–1.81	[9]
Targeted therapy	OlympiaA (NCT02032823)	III	Early II–III	Olaparib versus placebo in patients with *BRCA1/2* mutations	1509	DFS, distant: HR 0.57, 99.5% CI 0.39–0.83 OS: HR 0.68, 98.5% CI 0.47–0.97	[10,11]
Immunotherapy	SWOG S1418/BR-006 (NCT02954874)	III	Early II–III	Patients without pCR received pembrolizumab versus observation	Recruitment on going	Not yet reported	[12]

NCT, national clinical trial; DFS, disease-free survival; OS, overall survival; HR, hazard ratio; CI confidence interval; pCR, pathological complete response; BRCA, BReast CAncer gene.

**Table 2:** Neoadjuvant Triple Negative Breast Cancer TNBC curative-intent treatment.

	Trial (NCT Number)	Phase	Stage	Treatment	Patients in Analysis	Outcomes	Refs
Chemotherapy	GeparSixto (NCT01426880)	II	Early II–III	Neoadjuvant chemotherapy with versus without carboplatin	315	Achieving a pCR: OR 1.94, 95% CI 1.24–3.04	[13]
	CALGB 40603 (NCT00861705)	II	Early II–III	Weekly paclitaxel with versus without carboplatin, followed by AC	113	The addition of carboplatin increased pCR from 41% to 61%	[14]
	BrighTNess (NCT02032277)	III	Early II–III	Paclitaxel with versus without carboplatin	318	EFS: HR 0.57, 95% CI 0.36–0.91	[15,16]
Targeted therapy	I-SPY-2 (NCT02032277)	II	Early II–III	Paclitaxel with versus without veliparib–carboplatin	116	Rate of pCR: 51%, 95% CI 36–66 versus 26%, 95% CI 9–43	[17,18]
	BrighTNess (NCT02032277)	III	Early II–III	Paclitaxel and carboplatin with versus without veliparib	476	Rate of pCR: 53% versus 58% *p* = 0.36	[15,16]
	NEOTALA (NCT03499353)	II	Early I–III	Single-agent talazoparib in patients with *BRCA1/2* mutations	61	Rate of pCR: 49.2%, 95% CI 34.0–64.5	[19]
Immunotherapy	KEYNOTE-522 (NCT03036488)	III	Early II–III	Paclitaxel and carboplatin with versus without pembrolizumab, followed by AC	1174 -EFS 602 -pCR	EFS: HR 0.63, 95% CI 0.48–0.82 Rate of pCR: 64.8%, increase of 13.6%, 95% CI 5.4–21.8	[20]
	IMpassion031 (NCT03498716)	III	Early II–III	nab-paclitaxel followed by ddAC with versus without atezolizumab	333	Rate of pCR: 58%, increase of 17%, 95% CI 6–27	[21]
	GeparNUEVO (NCT02685059)	II	Early II–III	nab-paclitaxel followed by ddAC with versus without durvalumab	174	Rate of pCR: 53.4%, increase of 9.2%, NS Achieving a pCR OR 1.45, 95% CI 0.80–2.63 DFS: HR 0.54 OS: HR 0.26	[22]

NCT, national clinical trial; DFS, disease-free survival; OS, overall survival; EFS, event-free survival; NS, not significant; HR, hazard ratio; OR, odds ratio; CI confidence interval; pCR, pathological complete response; BRCA, BReast CAncer gene; AC, anthracycline + cyclophosphamide; ddAC, dose dense anthracycline + cyclophosphamide.

**Table 3:** Details of agents used in Triple Negative Breast Cancer (TNBC).

Agent Type	Agent Class	Agent	Mechanism	Relevant Trials	Ref
Chemotherapy	Anti-metabolites	Capecitabine	Prodrug that is converted to 5-FU, and subsequent metabolites inhibit formation of thymidylate, necessary for DNA synthesis	CREATE-X	[6]
	Alkylating agents	Platinum (carboplatin)	Reactive platinum complexes inhibit DNA synthesis by forming interstrand and intrastrand cross-linking of DNA molecules	GeparSixto	[13]
				CALGB 40603	[14]
				BrighTNess	[15,16]
		Cyclophosphamide	Prodrug that is metabolized to its active form phosphoramide mustard that forms cross-links between strands of DNA	ABC Trials	[5]
				CALGB 40603	[14]
				GeparNUEVO	[22]
	Anti-microtubule	Taxanes (paclitaxel, docetaxel)	Prevents effective microtubules by binding and promoting stabilization and growth	ABC Trials (See Table 1 and Table 2)	[5]
	Cytotoxic antibiotics	Doxorubicin	Intercalates with DNA leading to topoisomerase II inhibition and subsequent apoptosis	ABC Trials (See Table 1 and Table 2)	[5]
Targeted therapy	PARPi	Veliparib Talazoparib Olaparib	Inhibition of PARP leads to ineffective repair of DNA SSBs, leading to DSBs, and apoptosis	BrighTNess	[15,16]
				I-SPY-II	[17]
				NEOTALA	[19]
				OlympiaA	[10]
	AKTi	Ipatasertib	Inhibition of AKT slows down upregulated cell division pathways	FAIRLANE	[23]
Immunotherapy	PD-L1 inhibitor	Atezolizumab Durvalumab	MAB checkpoint inhibitor blocks PD-L1 interrupting the interaction with PD-1 on T-cells, enhancing antitumor immune response and leading to increased T-cell activation against tumors	IMPassion031	[21]
				GeparNUEVO	[22]
	PD-1 inhibitor	Pembrolizumab	MAB checkpoint inhibitor that blocks PD-1 as opposed to PD-L1, enhancing antitumor immune response and leading to increased T-cell activation against tumors	KEYNOTE522	[20]
Androgen Deprivation	Androgen receptor signaling inhibitor (ARSI)	Enzalutamide	Prevents the androgen receptor from translocating through the cell, preventing DNA transcription	MDV3100-11	[24]
	Pregnenolone Analogue	Abiraterone	Suppresses CPY17A1-mediated androgen synthesis and direct AR-inhibitory properties	UCBG 12-1	[24]
Antibody–Drug Conjugates		Sacituzumab govitecan-hziy	Antibody targeting Trop-2 linked to SN-38 (topoisomerase inhibitor), with chemotherapy released after cell internalization of the antibody.	NCT01631552	[25]
		Trastuzumab deruxtecan	Antibody targeting HER-2 linked to a topoisomerase inhibitor (deruxtecan). Used in patients with low expression of HER-2.	DESTINY-Breast04	[26]

5-FU, 5-fluorouracil; PARP, Poly-adenosine diphosphate-ribose polymerase; SSB, single-strand break; DSB, double-strand break; AKT, also called Protein kinase B; PD-L1, Programmed death-ligand 1; PD-1, Programmed death 1; MAB, monoclonal antibody; AR, androgen receptor; Trop-2, Human trophoblast cell-surface antigen 2; HER-2, Human epidermal growth factor receptor 2.
